# New Operative Method for the Radical and Rapid Cure of Chronic Empyemia of Maxillary Sinus

**Published:** 1898-03

**Authors:** George Randorf

**Affiliations:** Berlin, Germany.


ARTICLE IV.

### NEW OPERATIVE METHOD FOR THE RADICAL AND RAPID CURE OF CHRONIC EMPYEMIA OF MAXILLARY SINUS.

Reported by George Randorf, Berlin, Germany.

Mr. Luc, after having reminded us that the radical cure of the malady in question is one of the most difficult in rhinology, attributes the frequent failures of the different nasal and buccal operative procedures, to the former, allowing an insufficient means of reaching the seat of disease, and to the latter keeping this in too prolonged communication with the mouth, thereby exposing it to fresh infection. He has avoided the inconvenience of both these proceedings, and has united their advantages by the following method:

1. Free opening of the maxillary sinus, through incision of the gingival mucous membrane with the bistoury, at fifteen millimeters over the neck of the teeth, stamping of the mucous membrane with a rasp, and resection of the greatest part of the outer wall of the sinus;

2. Careful removal of all fungus in the sinus by means of electric cautery, and cauterizing with a strong solution of chloride of zinc;

3. Establishing, by means of chisel and mallet, of an orifice of communication between the sinus and the nasal cavity at the level of the lower part of the inner wall of the sinus;

4. Introduction of a drainage tube through the artificial opening; the tube is held in the cavity by its widened end, and emerges at the other end, through the nostril;

5. Reunion of the buccal wound by means of fine catgut, after the cavity has been well powdered with iodoform.

The buccal wound is cicatrized after three days, and the seat of the disease thus protected from the danger of buccal infection, is no longer in communication with the outer air except by the nose. During the following days the sinus is kept antiseptic by the drain, by means of injections of etherial iodoform, and irrigations with solutions of formal or boric acid. After a fortnight the drain is withdrawn from the nose by a slight traction, and the cure is complete.—*Items of Interest.*

